# Treatment of late solidified hematoma in back donor site after breast reconstruction with latissimus dorsi flap: report of three cases

**DOI:** 10.1186/s12893-019-0516-6

**Published:** 2019-05-20

**Authors:** Joon Seok Lee, Hyeon Jun Jeon, Jeeyeon Lee, Ho Yong Park, Jung Dug Yang

**Affiliations:** 1 Department of Plastic and Reconstructive Surgery, School of Medicine, Kyungpook National University, 130 Dongdeok-ro, Jung-gu, Daegu, 700-421 South Korea; 2 Department of Surgery, School of Medicine, Kyungpook National University, Hoguk-ro 807, Buk-gu, Daegu, 41404 South Korea

**Keywords:** Breast reconstruction, Latissimus dorsi flap, Chronic expanding hematoma, Solidified hematoma, Late hematoma

## Abstract

**Background:**

Late solidified hematoma is a rare complication of breast reconstruction with latissimus dorsi (LD) flap. The majority of hematomas occur in the immediate postoperative period; however, some cases can occur at a distant point in time after surgery and do not have a definitive mechanism of injury or develop symptoms immediately after the triggering event. Moreover, treatment of hematoma has not yet been established.

**Case presentation:**

Breast reconstruction with LD flap has been performed between January 2014 and June 2018 in more than 275 cases. We report 3 cases of late solidified hematoma at the back-donor site that have developed years after breast reconstruction with LD flap, in which a surgical approach was performed because the solidified hematomas could not be treated with percutaneous aspiration.

**Conclusions:**

We report successful surgical treatment and histological findings of late-onset solidified hematoma as a rare complication of Breast reconstruction with LD flap.

## Background

Chronic expanding hematoma (CEH) is a rare type of hematoma that occurs persistently and steadily without coagulation even after adequate recovery time after trauma or surgical intervention has passed. In general, hematomas that are small enough in size tend to dissolve steadily within the body as time passes, although this mechanism in the absence of known causes (i.e., trauma or surgeries) has not been fully understood. Previous reports show that CEH can occur even after months or years have passed since trauma or surgery [1–5]. In general, hematomas that occurred within few days after the surgery are usually observed during the hospitalization period, allowing the patient to receive aspiration and compressive therapy or surgical management through exploration in severe cases. However, the treatment of late hematoma that occurs months or years after the surgery is not well understood. Breast reconstruction with latissimus dorsi (LD) flap is commonly performed in breast cancer patients. Although previous studies described seroma as the most common complication in LD flap donor site, hematoma occurrence after multiple years since the surgery is rarely reported and late solidified hematoma that cannot be aspirated has never been reported before.

Sterling at el., Watanabe et al., and Brooker et al. suggested that CEH can occur when shearing force generated from stress damages the tissue and consequently induces bleeding, followed by poor coagulation [1–3]. Another report demonstrated that abnormalities in coagulation factors due to medication or underlying disease can also cause the onset of CEH [4].

CEH can occur in multiple areas of human body, and among them, in the back area, which is used in daily activities to move the body in different directions. Consequently, due to different reasons (trauma, breast reconstruction, cancer, etc.), there are frequent reports of CEH occurring on the LD donor site after reconstruction surgery using LD flap [2, 5, 6].

Although many previous reports on CEH have suggested different treatment methods, there is no standard treatment method, and evacuation through aspiration has been considered the most crucial aspect of the treatment [3, 6, 7]. In cases of persistent recurrence or incomplete remission with aspiration, operative approach is done for further treatment.

In this study, instead of focusing on seromas, which are the most common complication after immediate breast reconstruction using LD flap after mastectomy, we report 3 cases of late (≥18 months after the surgery) solidified hematoma of the LD flap donor site, which is very rare and cannot be treated with aspiration. In addition, we demonstrate treatment methods for these cases.

## Case presentation

During follow-up of 275 patients, out of 725 patients who underwent mastectomy and immediate breast reconstruction using LD flap between January 2014 and July 2018, we observed and selected 3 cases where the patient exhibited cystic mass suspected as late solidified hematoma that could not be treated with aspiration (Table 1). These 3 patients have developed hematoma in the form of a solidified mass at the LD donor site at 24, 48, and 18 months after breast reconstruction surgery. Axillary lymphadenectomy was executed for all 3 of the patients.

**Table 1:** Patient characteristics

NO.	Age (years)	BMI	Diagnosis	OP	Developed interval time (months)	Stage	RTx	CTx
1	59	23.2	DCIS, Lt.	TM + LD	24	1	–	–
2	41	25.5	IDC, Rt.	TM + LD	48	3	–	+
3	50	24	DCIS, Lt.	PM + LD	18	1	+	–

*BMI* body mass index, *OP* operation, *RTx* radiotherapy, *CTx* chemotherapy, *TM* total mastectomy, *PM* partial mastectomy, *Lt*. left, *Rt*. right

**Fig. 1:**
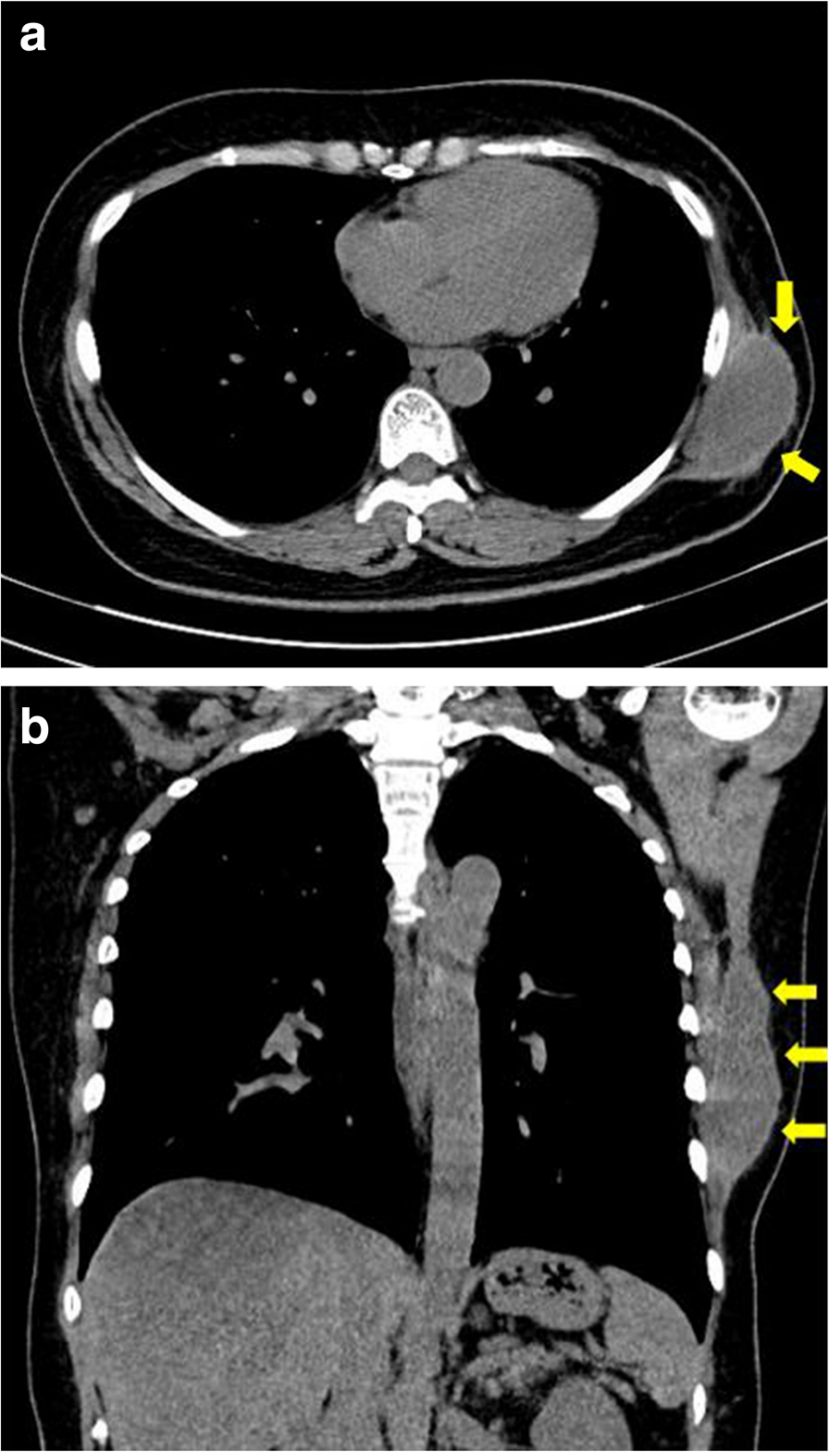
Preoperative computed tomography findings. Late solidified hematoma (yellow arrow) in the form of cystic mass (4 × 5 cm in size) is observed in the left LD donor site. (**a**) axial view, (**b**) coronal view

Treatment of complications (i.e., seroma, hematoma, etc.) at the LD flap donor site after breast reconstruction was immediate needle aspiration after confirmation of fluctuation under physical examination. After the aspiration, the amount of aspirate was recorded and compression dressing was applied using an elastic band. In case of drain of the LD flap after breast reconstruction, it is removed when it is under 30 for 2 consecutive days and the average removal period is around 2 weeks after the surgery. Other compression is not executed in the other LD donor site except for that. The regular follow-up takes place once a week for 2 weeks after the surgery, and the wound condition is checked out. Regarding physical activities, it occurs through rehabilitation medicine from a week after the surgery and it takes place through joint treatment with mild physical therapy in order to minimize the occurrence of a frozen shoulder. If quantitative improvement was not observed in 2 days after aspiration, re-aspiration was performed, followed by 40 mg triamcinolone/saline 1:1 mix injection (5 cc) and application of compressing dressing. Using this method, repetitive and multiple conservative treatments were performed. Lesions that persistently recur or remain solidified were first assessed for their pattern, area, and level under computed tomography (CT) (Fig. 1). Regardless of the part affected by mass, in case of the liquid mass, cytological evaluation is always carried out after execution if aspiration is available. In addition, hematoma and seroma are classified through Hct to take care of it. If a capsulated hematoma in the form of cystic mass was observed, the lesion was assessed under general anesthesia. Both solidified hematoma and capsule were entirely removed. To prevent recurrence of the resected lesion due to shearing force, quilting and bolster sutures were performed to compress the cavity completely. Follow-up was performed to monitor potential necrosis of the suture site. A negative pressure drain was positioned within the cavity, which was removed if &lt; 10 cc was observed over two consecutive days.

### Case 1

**Fig. 2:**
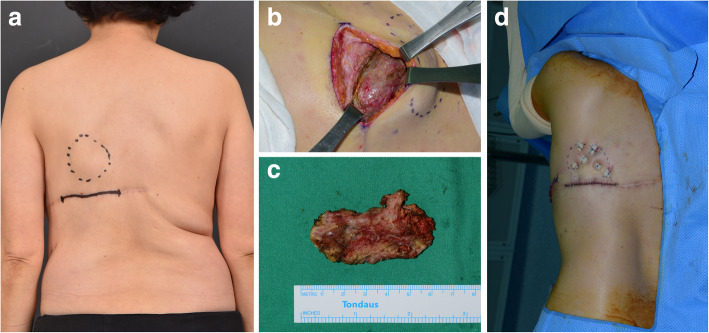
Case 1. (**a**) Preoperative findings. (**b**) Solidified hematoma findings located underneath the lower pole of the scapula. (**c**) Excised late solidified hematoma. (**d**) Immediate postoperative findings

A 59-year-old female patient (height, 146 cm; weight, 49 kg; body mass index [BMI], 23.2) had undergone left mastectomy for breast cancer (T1N0M0 stage 1) and immediate reconstruction surgery with an LD flap (Fig. 2). The patient visited our outpatient clinic 2 years after mastectomy, due to an acutely developed palpable mass at the back donor site. Physical examination results indicated the development of a solid mass at the location corresponding with the previous LD flap donor site (Fig. 2). The patient did not experience any precipitating event or blunt trauma and was not using medications with bleeding tendency (i.e., anticoagulant).

We initially tried to aspirate the palpable mass, but it could not be aspirated. Chest CT was performed to further assess the lesion, and a 3-cm, low-density lesion of late solidified hematoma in the form of cystic mass surrounded by capsular structure at the posterior aspect below the left scapula was confirmed.

As the lesion could not be removed via aspiration, surgical excision under general anesthesia was planned. During surgical excision, we observed a capsule-enveloped hematoma, and inside, a solidified hematoma with semisolid blood clots was identified. A definitive diagnosis was made based on the results of pathological examination. Biopsy revealed that the capsule consisted of fibrous tissue, and the content of the cyst comprised some blood and fibrinoid material. On day 6 after the surgery, the negative pressure drain was removed and the patient was discharged. During outpatient follow-up visits, seroma aspiration of the excised site was performed 4 times in total. The patient was followed up, and there was no recurrence or need for aspiration for 8 months.

### Case 2

**Fig. 3:**
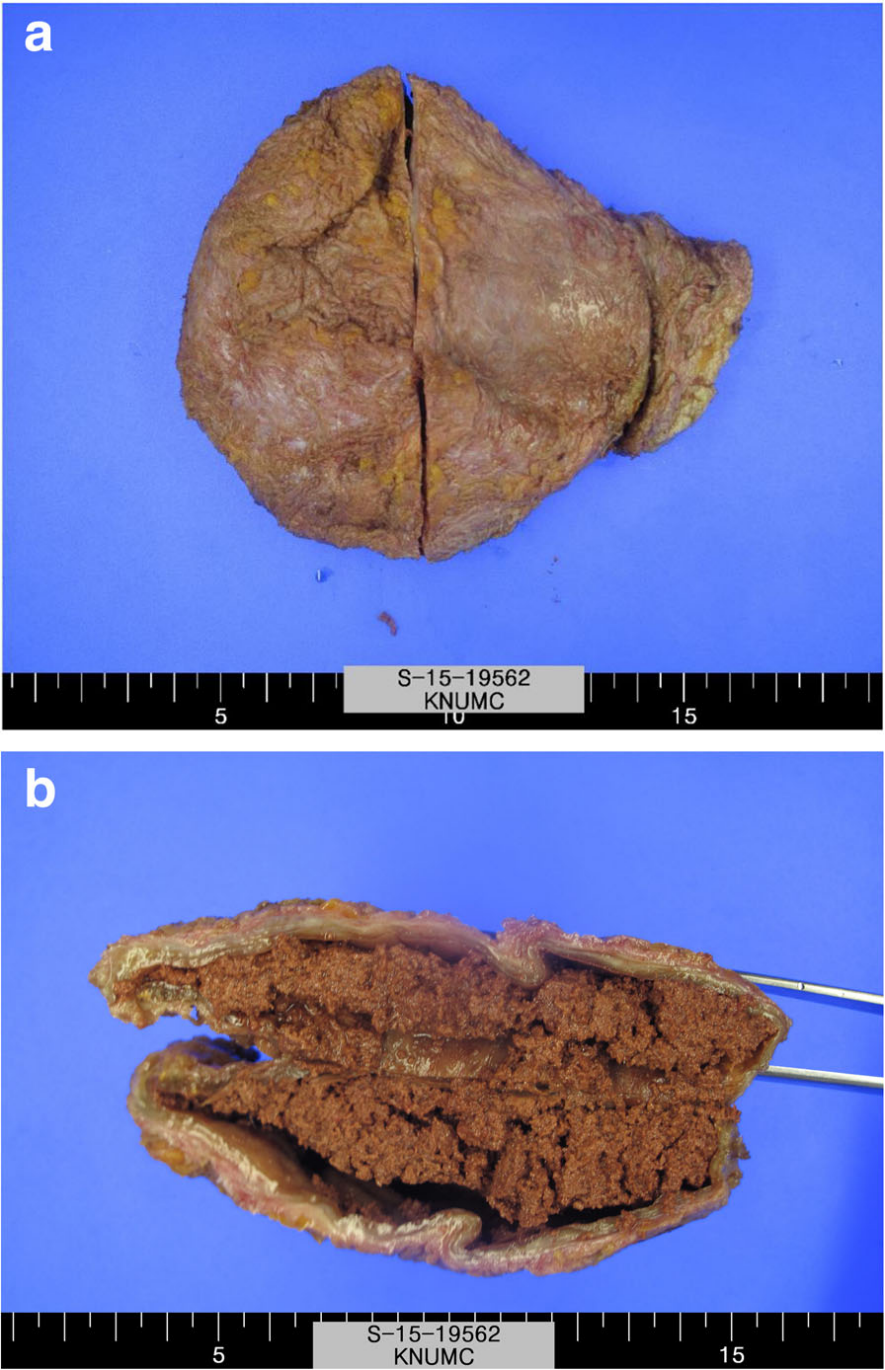
(**a**) Gross findings of the late solidified hematoma. (**b**) Dissected findings. The solidified hematoma was surrounded by a capsule and completely filled. The lesion cannot be aspirated

A 41-year-old female patient (height, 168 cm; weight, 72 kg; BMI, 25.5) had undergone right mastectomy for breast cancer (T2N2M0 stage 3) and immediate reconstruction surgery with an LD flap. After 4 years, the patient exhibited an acutely developed palpable mass at the back donor site and was examined at the surgery department in our center. Physical examination showed the development of a solid lesion that could not be aspirated. The patient did not have any specific triggering event or blunt trauma or any underlying diseases aside from uterine myoma and ovarian cyst. The patient was not under any medication. Chest CT confirmed the presence of an enlarged cystic mass (size 9 × 4 cm) in the right posterior chest wall, and surgical excision under general anesthesia was planned. During surgical excision, a capsule-enveloped hematoma was identified. A definitive diagnosis was made based on the results of pathological examination. Biopsy revealed no evidence of malignancy or benign cyst with fibrosis (Fig. 3). There was no recurrence or complication for 3 years.

### Case 3

**Fig. 4:**
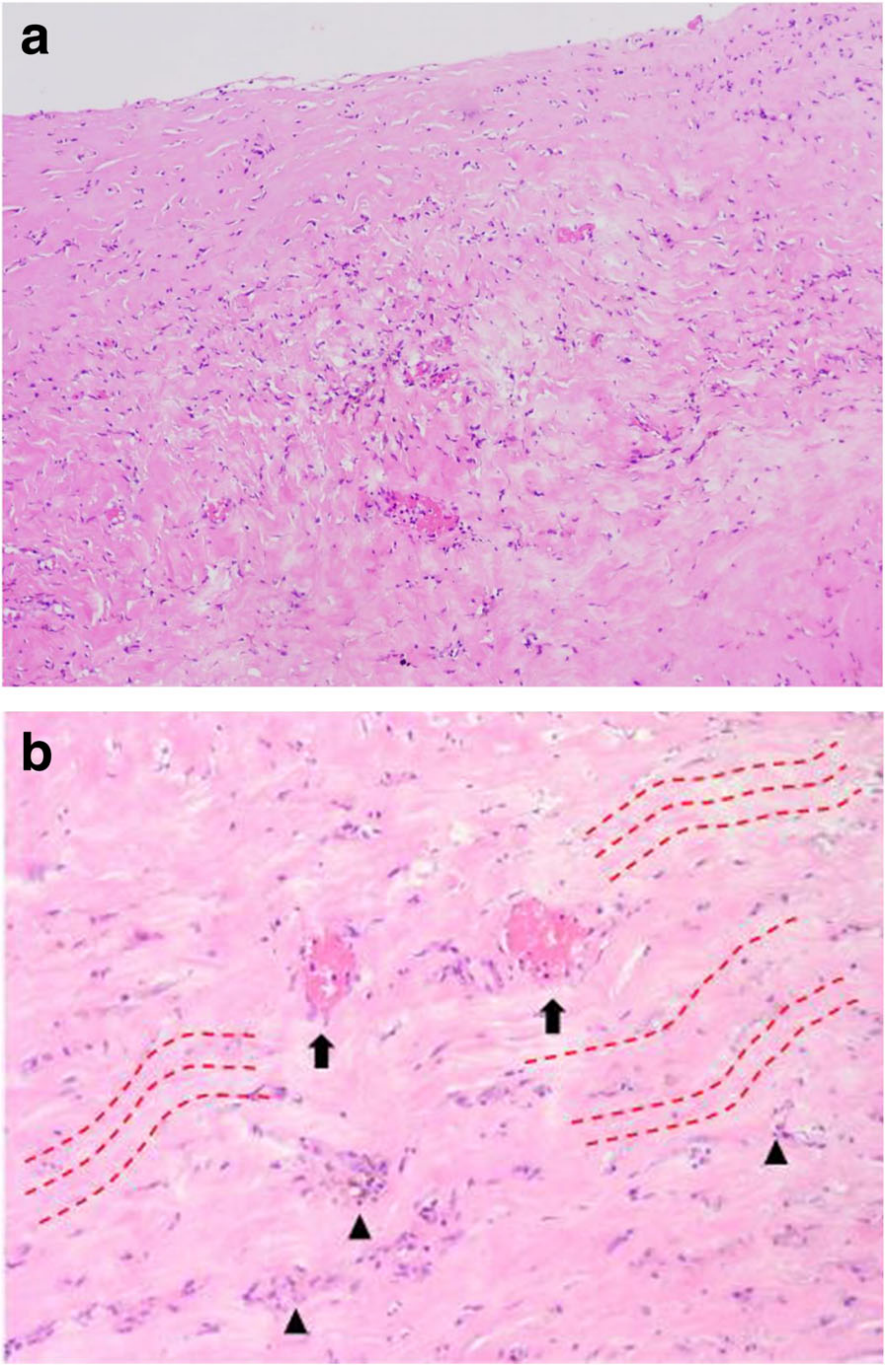
Histologic findings. Hematoxylin and eosin, × 100 (**a**) and × 200 (**b**). Dense fibrotic tissue (red dotted line) with some hemosiderin-laden macrophages and focal chronic inflammation (black arrowhead) and vessels (black arrow)

A 50-year-old female patient (height, 148 cm; weight, 53 kg, BMI, 24) underwent left partial mastectomy due to breast cancer (T1N0M0 stage 1) and immediate breast reconstruction surgery using an LD flap. The patient completed adjuvant radiotherapy and showed complete healing. However, 18 months after the breast reconstruction surgery, the patient visited our center with discomfort at the LD flap donor site. We observed a palpable mass resembling a solidified hematoma that could not be aspirated, and CT result confirmed the presence of a capsulated hematoma. Surgical excision under general anesthesia was planned, and both late solidified hematoma and capsule were removed using. Histologic examinations showed that the lesion was composed of dense fibrotic tissue, with accompanying focal chronic inflammation (Fig. 4).

## Discussion and conclusions

CEH can occur anywhere within the human body, due to multiple different reasons. Lewis and Johnson reported that hematoma development at the LD flap donor site is very rare [5, 6].

Although many previous studies reported on the various causes that induce hematoma, its exact etiology and mechanism of formation are not yet fully understood. Bradshaw et al. and Sterling et al. reported that shear stress-induced bleeding between the subcutaneous skin and the fascia can cause hematoma [1, 8]. In most cases, it was difficult for the patients to identify it due to the characteristic of the affected area, which is the back. A significant amount of time passed ever since it started out as a small size and grew bigger to a certain size and the patient could identify it. It is considered that during that period, signs for chronic alterations like blood vessels, fibrotic tissue and the capsular structure occurred. A previous study suggested that excessive activity can induce late hematoma at the LD flap donor site, but there were no abnormal precipitating factors in our cases. As late solidified gradually progressed, it was not discovered during the regular follow-up period, then once the patient identified it, they came in for treatment because of the first symptom that they had not noticed until then and received treatment for it. Therefore, we suspect that these shearing forces can be easily generated in the back region with daily activities, such as turning over during sleep or twisting the waist while walking. Moreover, another previous study on bleeding tendency demonstrated that anticoagulant medication (i.e., aspirin) can also have impact on the occurrence of late hematoma [2]. Nonetheless, the specific causes remain unclear, and therefore, prevention is very difficult. Regardless, patients who are currently using anticoagulant or who have experienced blunt trauma should be informed of potential late hematoma development, and clinicians should pay more attention to these individuals.

Regarding the duration from flap surgery to development of late hematoma, previous reports demonstrated that the development of late hematoma can occur anywhere between 1 month and 15 years after surgery [2, 3, 7]. In our cases, all 3 patients exhibited development of hematomas at least 18 months after surgery. Thus, surgeons should be aware that late hematomas can develop at the back donor site without any precipitating events in patients who have undergone breast reconstruction with LD flap several years after surgery, and surgeons should bear in mind that the duration between flap surgery and the development of CEH may range from months to years.

There are few reports on the treatments for old hematoma. Hamada et al. reported that 2 out of 3 patients received triamcinolone injection, and this treatment method can be considered over surgical excision or aspiration. However, similar to our cases, they performed surgical excision for the solidified hematoma, which could not be aspirated or injected with triamcinolone. Additionally, the most appropriate triamcinolone dose for CEH treatment, under triamcinolone injection protocol, should be assessed further with a larger number of cases [9].

In addition, based on the location and pattern of hematoma, other diseases (i.e., another soft tissue neoplasm) should also be considered. Therefore, additional CT and biopsy during surgery are required to assess this relationship. Deveci et al. reported that elastofibroma dorsi is a rare, benign, soft tissue tumor typically located between the inferior corner of the scapula and the posterior chest wall [10]. This tumor exhibits a similar location of onset and pattern as late hematoma, and therefore, differentiation between late hematoma and other soft tissue tumors should be performed. To ensure this differential diagnosis, radiologic examinations including ultrasound and CT, as well as additional postoperative pathological examinations should be performed. Before the intervention, the result showed that the particular mass was a cystic mass on the CT and it was the LD donor site instead of places where metastasis mostly occur such as bone, liver, lung, and brain. Even with what was shown with the CT scan, the possibility of metastatic cancer or remnant cancer was excluded and as for the place where seroma, which the major complication of LD flap surgery occurs more commonly, it can be suspected by priority that it is the hematoma that occurred on the surgical field based on the author’s medical and therapeutic experiences. The protocol for the treatment of hematoma is yet to be established. Nonetheless, in this case report, we demonstrated successful treatment in 3 cases of late-onset (≥18 months after breast reconstruction using an LD flap) solidified hematoma that occurred without known precipitating factors above the LD flap incision line (i.e., the region near the lower pole of the scapula) that could not be treated with aspiration.

## Abbreviations

BMI: Body mass index
CEH: Chronic expanding hematoma
CT: Computed tomography
LD: Latissimus dorsi
